# Comparison of Dual-Energy X-ray Absorptiometry and Bioelectrical Impedance Analysis in the Assessment of Body Composition in Women with Anorexia Nervosa upon Admission and Discharge from an Inpatient Specialist Unit

**DOI:** 10.3390/ijerph182111388

**Published:** 2021-10-29

**Authors:** Afrouz Abbaspour, Kylie K. Reed, Christopher Hübel, Emily C. Bulik-Sullivan, Quyen Tang, Cynthia M. Bulik, Ian M. Carroll

**Affiliations:** 1 Department of Medical Epidemiology and Biostatistics, Karolinska Institutet, 17177 Stockholm, Sweden; christopher.1.huebel@kcl.ac.uk (C.H.); cynthia_bulik@med.unc.edu (C.M.B.); 2 Department of Nutrition, Gillings School of Global Public Health, University of North Carolina at Chapel Hill, Chapel Hill, NC 27599, USA; reedkk@email.unc.edu (K.K.R.); emily_bulik-sullivan@med.unc.edu (E.C.B.-S.); ian_carroll@med.unc.edu (I.M.C.); 3 Social, Genetic & Developmental Psychiatry Centre, Institute of Psychiatry, Psychology & Neuroscience, King’s College London, London SE58AF, UK; 4 UK National Institute for Health Research (NIHR) Biomedical Research Centre for Mental Health, South London and Maudsley Hospital, London SE58AF, UK; 5 National Centre for Register-Based Research, Department of Economics and Business Economics, Aarhus University, 8000 Aarhus, Denmark; 6 School of Medicine, University of North Carolina at Chapel Hill, Chapel Hill, NC 27599, USA; 7 Graduate School of Professional Psychology, Morrison Family College of Health, University of St. Thomas, Minneapolis, MN 55403, USA; quyen.tang@stthomas.edu; 8 Department of Psychiatry, University of North Carolina at Chapel Hill, Chapel Hill, NC 27599, USA

**Keywords:** eating disorders, foot-to-foot impedance, limits of agreement, body mass index, re-nourishment

## Abstract

Assessment of body composition is fundamental in diagnosis and treatment of anorexia nervosa (AN). The gold standard dual-energy X-ray absorptiometry (DXA) is expensive and not universally available. Bioelectrical impedance analysis (BIA) is a non-invasive, inexpensive method relative to DXA. We compared DXA and BIA in the assessment of fat-free mass (FFM), fat mass (FM), and body fat percentage (BF%) in women with AN upon admission (ANT1) and discharge (ANT2) from an inpatient specialist unit with a referent healthy control (HC) group. The study population consisted of 31 ANT1, 25 ANT2, and 52 HC women with median age of 21 years. Body composition was measured by DXA and Tanita foot-to-foot BIA. Comparison between the two methods was done using Bland–Altman analysis, Pearson’s correlation coefficient, Lin’s concordance correlation coefficient, and linear regression. The mean difference (bias) in FM and BF% values obtained by DXA and BIA in ANT1 (FM: +1.01 kg, BF%: +2.26%) and ANT2 (FM: +1.49 kg, BF%: +1.66%) were comparable to HC (FM: −1.32 kg, BF%: −2.29%) although in opposite directions. Less bias was observed in FFM values in ANT1 (−0.46 kg) and ANT2 (−0.86 kg) than in HC (+2.03 kg); however, the limits of agreement between the two methods were wider in ANT1 and ANT2 than in HC for all measurements. No association was observed between age, percentage of total body water, and the time spent on the inpatient specialist unit with the difference in estimates of body composition between DXA and BIA. Comparison of DXA and BIA suggests that DXA should remain the gold standard for measuring body composition; the development of more specific BIA equations is required to improve validity and precision of BIA in patients with AN. Despite ease and cost in both BIA access and operation, the suitability of BIA in a low bodyweight eating disorders population remains questionable.

## 1. Introduction

Anorexia nervosa (AN) exhibits a high mortality rate [1,2] and is characterized by dangerously low bodyweight, indifference to the seriousness of the illness, and female preponderance [3]. The average lifetime prevalence of AN is estimated to be 1.4% for women and 0.2% for men [4]. Undernutrition in AN leads to alterations in body composition [5]; consequently, accurate assessment of body compartments plays an integral role in evaluating disease status and treatment progression [6].

Accurate assessment of body composition in individuals with AN is crucial for developing and monitoring nutritional rehabilitation interventions, addressing coexisting medical conditions, and selecting appropriate treatments. Clinically relevant changes in body composition may vary among patients with AN due to differences in the manifestation of the eating disorder, specifically regarding food restriction, vomiting, excessive exercise, laxative use, and water intake [7]. Although the magnitude of changes may vary by sex and age [5], reductions in both fat-free mass (FFM) and fat mass (FM) have been observed [8]. Preferential loss of body fat has been reported in AN [9], inferring that reductions in fat mass account for a large portion of alterations in body composition in individuals with this condition. Additionally, unstable total body water (TBW) is a common characteristic of individuals with AN, pointing to marked dehydration upon admission to treatment [10].

Bodyweight and BMI alone are not adequately sensitive to determine nutritional status in a population with severe malnutrition because they do not reflect changes in body compartments such as FFM and FM [7,11,12]. Previous studies have shown that BMI only explains ~20–50% of the variance in body fat percentage measured by skinfold anthropometry, densitometry, or dual x-ray absorptiometry (DXA) [12,13,14,15]. Despite the role of BMI in the diagnosis of AN [3], several clinical methods are available to monitor specific alterations in body composition more closely. A pixel-based whole body imaging technology, DXA is a reliable and valid tool for measuring body composition and uses the 3-compartment chemical model of body composition [16]. DXA uses two different X-ray energy levels to measure bone mineral density and total body composition as the primary variables. Subsequent exclusion of bone tissue from lean mass values allows for the quantification of FFM and FM. However, the utility of and access to DXA is challenged by the fact that it is expensive, not universally available, requires a skilled operator, and emits low levels of radiation to the patient.

Bioelectrical impedance analysis (BIA) is a more simplified technique that requires less operator training [17] and is markedly more affordable [18]. BIA calculates FFM using a 2-compartment chemical model of body composition [16] and mathematical equations [7] that rely on the assumption that hydration status is constant. FM is then calculated indirectly by subtraction of the FFM from the total bodyweight [19,20]. Because hydration status and TBW are not constant in AN [18,21], the validity of this technique remains questioned. Few studies have evaluated the accuracy of BIA in a malnourished population [6,7,8,17,18,21,22,23,24,25], and the application of BIA and the related equations in AN populations requires further validation. Additionally, the majority of these limited studies employ the use of hand-to-foot BIA systems, transmitting and receiving electric signals through sites at the upper and lower extremities [26]. Foot-to-foot BIA systems simplify body composition measurements by eliminating the need for gel electrodes; patients simply step onto the digital scale with bare feet to make pressure contact with the indefinitely reusable electrodes and transmit a signal. Foot-to-foot BIA models operate without fluid displacement which enhances their rapidity of the measurements compared with hand-to-foot systems. Furthermore, high correlation has been reported between impedance derived from hand-to-foot BIA models and impedance obtained from foot-to-foot systems suggesting they can be tested for clinical application [27]. To our knowledge, no studies have used the foot-to-foot impedance model nor evaluated the validity of this technique after therapeutic renourishment in AN patients, in which TBW would be expected to normalize towards the levels observed in healthy individuals. Indeed, body fat distribution and TBW are known to normalize after weight restoration [9]; therefore, BIA potentially represents a better approach for body composition assessment after therapeutic renourishment in AN.

We evaluated the validity of BIA compared with DXA in a sample of women with AN before and after inpatient renourishment.

## 2. Materials and Methods

A total of 31 female patients with AN and 52 healthy control (HC) individuals between the age of 15 to 38 years were included in the study. Patients were recruited from the Center of Excellence for Eating Disorders (CEED) at the University of North Carolina (UNC) who presented for inpatient treatment at <75% of ideal bodyweight. AN diagnosis was established via the Eating Disorders Examination [28] and the eating disorders module of research version of the Structured Clinical Interview for DSM-5 (SCID-5) [29]. All measurements were recorded for patients with AN at baseline (i.e., between 1–15 days [mean 4 days] following informed consent) (ANT1, *n* = 31) and at discharge (ANT2, *n* = 25). The range for duration of inpatient treatment was 3 to 75 days with a median of 21 days. Age-matched female controls were recruited from university listservs and university-supported research recruitment warehouses. The inclusion criteria for healthy controls were stable weight (BMI 18.5–24.9 kg/m^2^), regular menstruation, no history of eating disorders or other psychiatric history, and no history of an adult BMI outside of the 18.5–24.9 kg/m^2^ range. All participants were instructed to fast for at least 30 min before the assessment of body composition. This study was part of a parent study investigating the functional influence of intestinal microbiota on adiposity and behavioral traits associated with AN. All participants provided informed consent and the study was approved by the UNC Biomedical Institutional Review Board (IRB#: 15-2133). Flow diagram for the study participants is presented in Figure S1.

### 2.1. Anthropometry

Bodyweight and height were measured using a calibrated digital scale and a stadiometer. BMI was calculated as weight divided by square of height (kg/m^2^).

### 2.2. Dual-Energy X-ray Absorptiometry (DXA)

DXA measurements of body composition were performed in ANT1, ANT2, and HC using the Hologic^®^ Discover-W system (Hologic Canada ULC, Mississauga, ON, Canada). The total and regional body mass measurements consisting of fat mass (FM), fat-free mass (FFM), and bone mineral content (BMC) were obtained. Body fat distribution was determined using the method published by Mayer et al. [30] that calculates extremity fat as a percentage of total fat (extremity fat mass/total fat mass) and trunk (central body) fat.

### 2.3. Bioelectrical Impedance Analysis (BIA)

BIA measurements of body composition were performed using the standing foot-to-foot Tanita DC-430U^®^ machine (Tanita Corporation, Tokyo, Japan) on the same day as DXA scans were obtained for the majority of the participants (68% of participants). When measurements on the same day were not possible, BIA was performed one day after DXA (32% of participants). Participants were asked to remove their clothes to wear a hospital gown and remain in a standing position before stepping on the instrument. They were then instructed to stand on the scale with bare feet in a wide and comfortable stance with their arms relaxed and to their side, looking forward and remaining still and as relaxed as possible. It is important to note that because gravitational forces will have a stronger effect on the measurement when in a standing position, a subject’s standing position may change as both time of measurement and condition of the subject vary [31]. A consistent amount was subtracted from the weight measurements to account for the weight of the hospital gowns. Resistance (R) and Reactance (Xc) in Ohm were recorded at two frequencies (6.25 and 50 kHz) while only 50 kHz records were used for further calculations. The FM was estimated using the software provided by the manufacturer of the bioelectrical impedance analyzer (Tanita HealthWare^®^ Native Software, Tokyo, Japan). FFM was then calculated indirectly by subtraction of the FM from the total bodyweight.

### 2.4. Statistical Analysis

Statistical analysis was performed using open-source R v3.5.1 (Vienna, Austria) and RStudio v1.1.463 (Boston, MA, USA) software packages (r-project.org) including blandr v.0.5.1 package (https://github.com/deepankardatta/blandr, accessed on 10 October 2020) to carry out Bland-Altman analyses. Results are expressed as median interquartile ranges (IQR) since the data are not normally distributed. To assess whether the BIA measures are clinically and physiologically relevant, we estimated the percentage of patients with measures above min values obtained from a meta-analysis of body composition in patients with AN before and after treatment [5]. Correlation and concordance analyses between DXA and BIA measures of body composition was performed in ANT1, ANT2, and HC using Pearson’s correlation coefficient and concordance correlation coefficient of Lin [32]. In order to determine agreement between the two methods, Bland–Altman analysis [33] with calculation of the 95% limits of agreement was performed. Linear regression was used to assess whether the difference in estimates of body composition between DXA and BIA is associated with age, TBW%, or the time spent on the inpatient specialist unit in each group. The differences in estimates of body composition between DXA and BIA (ΔFFM, ΔFM, and ΔBF%) were used as dependent variables. Age, TBW%, and the time spent on the inpatient specialist unit were used as predictors. The association between the length of time spent on the inpatient specialist unit and the changes in body composition measures from T1 to T2 was evaluated using regression analysis. A statistical significance was considered when *p* < 0.05.

## 3. Results

### 3.1. Descriptive Statistics of the Sample Population

In total, 31 patients with AN at baseline (ANT1), 25 at discharge from an inpatient specialist unit (ANT2), and 52 healthy control (HC) individuals were included in the study. All participants were female. Table 1 summarizes the characteristics of the sample. Briefly, study participants had median age of 21 years and comparable heights in all groups. ANT1 group had the lowest median BMI (16.6 kg/m^2^) followed by ANT2 (18.5 kg/m^2^) and HC (21.8 kg/m^2^). Regardless of the assessment method, all body compositions measures (FFM, FM, and BF%) were lower in ANT1 compared with ANT2 and HC. The measures increased in the ANT2 group at discharge, but not to the same level as in the HC group. Figure S2 visualizes the body composition measures obtained from DXA and BIA as box plots.

### 3.2. Comparison of DXA and BIA Estimates of Body Composition

**Figure 1:**
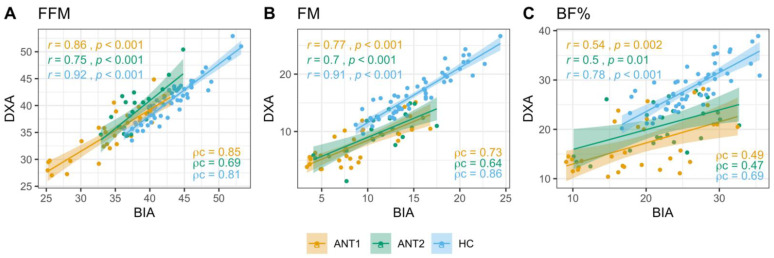
Correlation and concordance between DXA and BIA estimates of body composition. Strength of relation between DXA and BIA derived FFM (**A**), FM (**B**), and BF% (**C**) in ANT1, ANT2, and HC was assessed using the Pearson’s and Lin’s concordance correlation coefficients. *r*: Pearson’s correlation coefficients, *p*: *p* values, ρc: Lin’s concordance correlation coefficient. FFM: Fat-free mass, FM: Fat mass, BF%: Body fat percentage.

**Figure 2:**
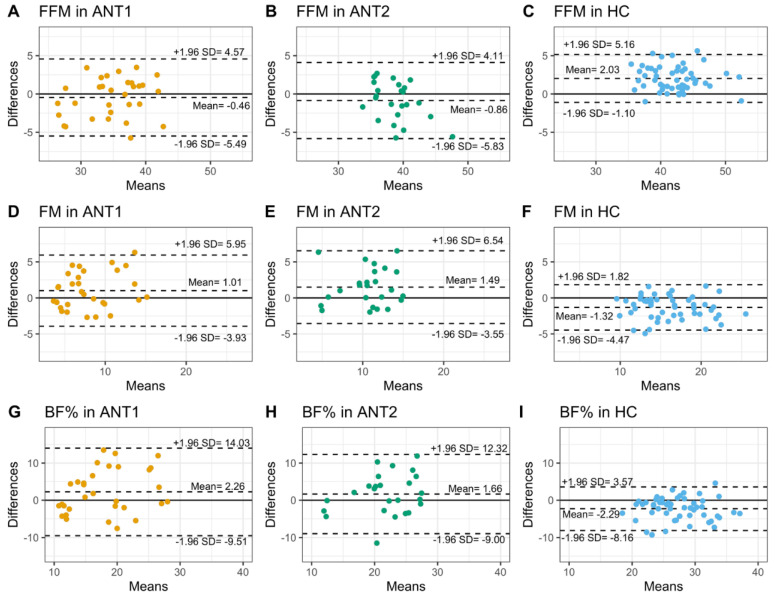
Bland-Altman agreement between DXA and BIA estimates of body composition. The agreement between DXA and BIA derived body compositions was assessed using Bland-Altman analysis. Mean bias and limits of agreement are shown as dotted lines in ANT1 (orange), ANT2 (green), and HC (blue), for FFM (**A**–**C**), FM (**D**–**F**), and BF% (**G**–**I**). FFM: Fat-free mass, FM: Fat mass, BF%: Body fat percentage, DXA: Dual-energy X-ray absorptiometry, BIA: Bioelectrical impedance analysis.

The proportion of individuals with AN with body composition measures above the defined clinically relevant minimum values [5] were estimated for the BIA method (Table S1). Results from Pearson’s correlation coefficient and concordance correlation coefficient of Lin (Figure 1, Table S2) and Bland-Altman agreement analyses of body composition measures derived from DXA as the reference method and the same measures estimated by BIA (Figure 2, Table S2) are presented.

#### 3.2.1. Fat-Free Mass

The proportion of ANT1 and ANT2 patients with BIA measures above the defined minimum values (ANT1 = 28.4 kg, ANT2 = 36.2 kg) was greater than 80% (Table S1). FFM measures obtained from BIA showed higher correlations and concordance with DXA measures in HC (*r* = 0.92, Pearson; ρc = 0.81, Lin) and ANT1 (*r* = 0.86, Pearson; ρc = 0.85, Lin) than in ANT2 (*r* = 0.75, Pearson; ρc = 0.69, Lin, Figure 1A, Table S2). BIA showed a robust agreement with DXA in both ANT1 and ANT2, with a marginal underestimation of FFM by 0.46 and 0.86 kg, respectively. The BIA overestimated the FFM by 2.03 kg in HC but the area of variation between upper and lower limits of agreement between DXA and BIA was smaller than those in ANT1 and ANT2 (Figure 2A–C, Table S2).

#### 3.2.2. Fat Mass

All patients at ANT1 and 88% of patients at ANT2 had FM measures above the literature-based defined minimum values (ANT1 = 0.5 kg, ANT2 = 7.2 kg, Table S1). FM measures obtained from BIA and DXA showed higher correlation and concordance in HC (*r* = 0.91, Pearson; ρc = 0.86, Lin) than in ANT1 (*r* = 0.77, Pearson; ρc = 0.73, Lin) and ANT2 (*r* = 0.7, Pearson; ρc = 0.64, Lin, Figure 1B, Table S2). The Bland-Altman agreement analysis indicated an overestimation of FM by BIA relative to DXA in both ANT1 (mean bias = 1.01 kg) and ANT2 (mean bias = 1.49 kg). In contrast, BIA underestimated FM measures by 1.32 kg in HC but with a smaller area of variation between upper and lower limits of agreement than ANT1 and ANT2 (Figure 2D–F, Table S2).

#### 3.2.3. Body Fat Percentage

All patients at ANT1 and 84% of patients at ANT2 had BF% measures above the literature-based defined minimum values (ANT1 = 2.4%, ANT2 = 17.5%, Table S1). The correlation and concordance between BIA and DXA estimates of BF% were satisfactory in HC (*r* = 0.78 Pearson; ρc = 0.69, Lin) but poor in ANT1 (*r* = 0.54, ρc = 0.49, Lin) and ANT2 (*r* = 0.5, ρc = 0.47, Lin, Figure 1C, Table S2). The Bland-Altman agreement analysis of BF% indicated trends similar to those observed in FM. BIA overestimated BF% measures in ANT1 (mean bias = 2.26 % points) and ANT2 (mean bias = 1.66% points) and underestimated BF% in HC (mean bias = −2.29 % points), with HC showing a smaller area of variation between upper and lower limits of agreement than ANT1 and ANT2 (Figure 2G–I, Table S2).

Bland-Altman plots also revealed that the differences of FFM and FM measured by the two methods changed depending on the mean. Mean bias of FFM between DXA and BIA methods was negative for lower values (observed in ANT1 and ANT2) and positive for higher values (observed in HC). By contrast, mean bias of FM between the two methods was positive for lower values (observed in ANT1 and ANT2) and negative for higher values (observed in HC). Existence of a proportional bias was further confirmed for FFM and FM by regressing the differences between the two methods on the average of the two methods (Figure S3). No relationship was observed between differences and means of BF% derived from DXA and BIA (data not shown).

### 3.3. Associations of Age, Percentage of Total Body Water, and Time Spent on the Inpatient Specialist Unit with the Difference in Estimates of Body Composition between DXA and BIA

We performed multiple linear regression to assess whether the difference (delta, Δ) in estimates of body composition between DXA and BIA was associated with age, TBW%, or the time spent on the inpatient specialist unit in each group. Differences in estimates of body composition between DXA and BIA **(**ΔFFM, ΔFM, and ΔBF%) were used as dependent variables. In Model 1, age and TBW% were used as independent variables. In Model 2, time spent on the inpatient unit was added as an additional independent variable. Because time spent in the unit is only relevant in ANT2, Model 2 was only tested in this group. Table 2 shows the absence of association between delta of measurements with age, TBW%, and the time spent on the inpatient specialist unit.

### 3.4. Associations between Time Spent on the Inpatient Specialist Unit and the Changes in DXA and BIA Estimates of Body Composition from T1 to T2

In order to evaluate the associations between time spent on the inpatient specialist unit and the changes from T1 to T2 in DXA and BIA derived body composition measures, we performed simple linear regression analysis. The length of time spent on the inpatient specialist unit explained 35–45% of the variation observed in FFM and FM measures obtained from DXA and BIA. The length of time spent on the inpatient specialist unit showed a more robust association with changes in BF% obtained from DXA (*r^2^* = 0.73) than from BIA (*r^2^* = 0.26, Table 3).

We also performed linear regression to evaluate the associations between DXA and BIA derived changes in estimates of body composition from T1 to T2 (Table S3). Our results indicate that a stronger association exists between DXA and BIA derived FFM_(T2–T1)_ and FM_(T2–T1)_ (*r*^2^ = 0.63 and 0.67, respectively) than BF%_(T2–T1)_ (*r*^2^ = 0.24). When age, TBW%, and time spent on the unit were added to the model as covariates, the adjusted correlation coefficient of determination increased for BF%_(T2–T1)_ (*r*^2^ = 0.84), but the variation in DXA BF%_(T2–T1)_ was explained by age and time spent on the unit rather than by BIA BF%_(T2–T1)_.

## 4. Discussion

Due to the impact of AN on body composition, our study found lower FFM, FM, and BF% in patients with this disorder compared to the HC group—even after discharge from an inpatient specialist unit. With the exception of BF% measured by DXA, duration in the inpatient unit only accounted for a low percentage of the variation. Failure to regain normal levels of FFM, FM, and BF% in this patient population is consistent with the suggestion that a longer period of renourishment may be needed to properly mitigate undernutrition [34,35].

The measures of FM, FFM, and BF% in this population of female patients with AN depict mean difference (bias) between DXA and BIA comparable to healthy individuals; however, concerning limits of agreement between the two methods were observed. In addition, the agreement between the two methods was not equal through the range of FM and FFM measurements suggesting the existence of a proportional bias. Similar changes in differences between DXA and BIA estimates of FFM and FM according to the average have been previously reported [36], and on the population level, concordance between DXA and BIA suggests that the nature of this trend is of little clinical relevance. Correlations between DXA and BIA within the control group confirms BIA as a valid tool in healthy female population; however, the strength of the correlation and concordance within the ANT1 and ANT2 groups varied depending on the body composition variable with body fat percentage showing weaker correlation and concordance than FM and FFM. Inconsistent, varying correlations between DXA and BIA in AN are important to consider clinically, as a change in only a few kilograms at such low bodyweight corresponds to a higher relative error and may have significant implications both at admission and discharge from treatment [8]. The complications we found associated with BIA are at least in part due to the low body fat in patients at admission to treatment. Therefore, DXA may remain the preferred methodology until a patient’s fat mass increases to a healthier level after therapeutic renourishment [18]. Additionally, we speculated that dehydration in patients with AN at admission would also contribute to less accurate estimates of fat mass with BIA. BIA operates by sending an electrical impulse throughout the individual’s body and is largely dependent on body water levels [22]. Based on our data, TBW% did not affect differences between DXA and BIA. These results align with other studies reporting no significant effect of hydration on body composition DXA measurements within both normal-weight and AN patients [10,21,37]. When used with caution, BIA may provide relevant information about changes in body composition. However, its limitations as a cross-sectional measure in AN, especially at low bodyweight, should be considered.

The BIA native equation (Tanita) exhibited small bias compared with DXA measurements in AN patients, but the associated larger limits of agreement than HC confirms that discrepancies remain. Although data collected from the BIA equations provide body composition data that may aid in clinical assessment [7], the values recorded still do not provide an adequate depiction of FFM, FM, and BF% in AN patients. It is unclear why the mean biases in ANT1 and AN2 data are smaller compared with HC group. However, this might suggest that additional datapoints that are not accessible to the user or an additional part of the system equation used a “correcting” factor for bodyweights that vary outside of the norm. It will be important to develop a prediction model for the low bodyweight patient population that considers all aspects of the disease.

The Tanita equipment with stainless steel contact electrodes conducted in a standing position represents an adapted technique that replaced the traditional electrode measurements in a supine position. The application of electrical data from the Tanita foot-to-foot model to classical equations developed using hand-to-foot BIA instruments described in the literature [7] would be invalid; however, early validation studies reported that foot-to-foot BIA systems have performance similar to conventional hand-to-foot gel electrode BIA analyses conducted in the supine position, in conjunction with heightened ease and speed of measurement [26]. More recently, foot-to-foot BIA has been validated in healthy Asian individuals as a tool to predict FFM amongst participants with different BF% [38]. In their study population (BMI range 15.9–43.1 kg/m^2^), BIA-modeled FFM values did not significantly differ from FFM measured via DXA among BF% subgroups. Despite consistent accuracy across groups in this study, many studies have reported that BIA estimation may be influenced by the degree of obesity of the individual [39,40,41]. Thus, it is important to consider that the severity of physical (specifically, weight) perturbations induced by AN may disturb foot-to-foot BIA systems from measuring body composition accurately—and call for cautious interpretation.

After renourishment and weight restoration, body fat distribution and TBW normalize [9] in patients with AN. As existing BIA equations were developed in populations with healthy BMIs, and a corresponding range of FM, FFM, and BF% levels, we suspect that BIA may be more accurate for patients with AN following clinical renourishment. Our data show only small differences in the overall correlation and agreement between DXA and BIA at T2 compared to T1. This inability to identify large inconsistencies in accuracy over time indicates that, in an AN population, the Tanita equation functions similarly at both T1 and T2. It is important to recognize, however, that these findings may be affected by the small sample size, because ANT1 and ANT2 data included 25 patients in total. Another possible explanation for consistency across time points may be that the period from admission to discharge was not adequately long to result in substantial increases in body fat—underscoring the importance of adequate duration of treatment for AN.

It is important to note that our study had several limitations. This study has a small sample size that may reduce statistical power and increase error. Future studies should include larger sample sizes while maintaining the longitudinal assessment of body composition. Additionally, only one specific type of DXA and BIA apparatus with an unknown proprietary equation was used, and there may be variations between the equipment used for this study and the manufacturers of other machines. This may substantially impact the agreement between DXA and BIA measures. The length of fast before assessment may also contribute to variations in the data, but fasting information was not explicitly included in data collection to be accounted for in analysis. Finally, discrepancies between HC and AN patients may have skewed the results because HC were not BMI-matched with ANT2; however, BMI does not always fully normalize in AN patients undergoing inpatient treatment. In order to have a healthy BMI-matched control group, we would have to consider other populations such as constitutionally thin individuals.

According to our results, DXA should remain the gold standard for body composition assessment in patients with AN; wide Bland-Altman limits of agreement and weak Pearson’s and Lin’s concordance correlation coefficients suggest that foot-to-foot BIA using the native Tanita equation may not be a suitable option for individuals in a very low bodyweight population. Despite the consideration of clinical feasibility, our data do not concordantly lead to the recommended use of the Tanita equation at either timepoint (admission and discharge) to accurately measure body composition in patients with AN. Due to wide 95% confidence intervals and large changes in measurements according to the mean observed in Bland-Altman agreement analysis in this study, future research should further validate the use of BIA in the AN patient population and compare the Tanita equation to other native BIA software equations. The development of a new equation specific to AN may be necessary to optimize the use of BIA by increasing precision and validity.

## 5. Conclusions

Specific measurement of body composition in patients with AN provides information critical to both the provider and patient throughout the duration of illness. Bodyweight and BMI alone are not sufficient to comprehensively design and implement treatment in a clinical setting.

DXA remains the gold standard to assess body composition in patients with AN at admission and after therapeutic renourishment. Comparison of DXA and BIA suggests that in order to implement BIA as a sufficient tool to analyze body composition in patients with AN, the development of more specific equations is required to improve validity and precision. Despite ease and cost in both access and operation, the suitability of BIA in low bodyweight populations remains questionable.

Further research is needed to confirm not only the possibility of using BIA in patients with AN, but also to investigate and monitor shifts in body composition that may directly determine patient health status.

## Figures and Tables

**Table 1:** Descriptive statistics of the sample.

	ANT1	ANT2	HC
	(*n* = 31)	(*n* = 25)	(*n* = 52)
Age (years)	21.0 (9.50)	21.0 (10.0)	21.0 (6.00)
Height (cm)	163 (6.20)	164 (6.00)	164 (7.25)
Weight (kg)	43.5 (10.9)	50.4 (5.40)	59.2 (7.60)
BMI (kg/m^2^)	16.6 (3.30)	18.5 (1.30)	21.8 (2.73)
DXA			
FFM (kg)	35.8 (6.07)	39.5 (4.65)	41.2 (5.05)
FM (kg)	6.28 (5.33)	10.4 (3.72)	16.0 (5.80)
BF (%)	14.5 (8.50)	22.0 (8.10)	28.0 (6.25)
BIA			
Resistance: R50 (Ohm)	502 (153)	494 (112)	535 (80.7)
Reactance: Xc50 (Ohm)	28.9 (20.2)	35.5 (9.90)	46.5 (7.78)
FFM (kg)	34.9 (5.87)	37.7 (3.62)	43.2 (4.56)
FM (kg)	7.70 (4.50)	11.6 (3.40)	14.5 (5.53)
BF (%)	18.9 (9.60)	23.1 (6.30)	25.6 (5.80)
TBW (%) *	63.6 (10.4)	61.5 (6.00)	53.8 (4.38)

Values are expressed as median (IQR). Fat-free mass (FFM), fat mass (FM), and body fat percentage (BF%) were assessed by dual-energy X-ray absorptiometry (DXA) and bioelectrical impedance analysis (BIA). BIA resistance and reactance were measured at 50 kHz and the body composition measure were estimated using the Tanita native equation. BMI: Body mass index, IQR: Interquartile range. * Number of participants with recorded TBW %: ANT1 = 15, ANT2 = 15, HC = 52.

**Table 2:** Association of age, total body water percentage, and time spent on the inpatient specialist unit with the difference (delta, Δ) in DXA and BIA derived body composition measures.

Model	Group	Independent Variables	Dependent Variables					
			ΔFFM (kg)		ΔFM (kg)		ΔBF%	
			*β* (95% CI)	Total Adjusted *r*^2^	*β* (95% CI)	Total Adjusted *r*^2^	*β* (95% CI)	Total Adjusted *r*^2^
1	ANT1	Age	−0.09 (−0.25, 0.06)	0.06	0.08 (−0.07, 0.24)	0.06	0.22 (−0.11, 0.55)	0.10
		TBW%	0.09 (−0.07, 0.25)		−0.10 (−0.26, 0.07)		−0.20 (−0.55, 0.15)	
	ANT2	Age	0.11 (−0.08, 0.29)	−0.003	−0.11 (−0.30, 0.08)	0.01	−0.07 (−0.52, 0.39)	−0.14
		TBW%	0.05 (−0.20, 0.29)		−0.05 (−0.31, 0.20)		−0.09 (−0.69, 0.51)	
	HC	Age	0.01 (−0.12, 0.13)	−0.04	−0.01 (−0.13, 0.12)	−0.04	0.03 (−0.20, 0.26)	−0.02
		TBW%	−0.01 (−0.17, 0.15)		0.00 (−0.16, 0.16)		−0.12 (−0.41, 0.18)	
2	ANT2	Age	0.15 (−0.06, 0.36)	−0.01	−0.16 (−0.37, 0.06)	0.01	0.00 (−0.53, 0.54)	−0.20
		TBW%	0.02 (−0.24, 0.27)		−0.02 (−0.29, 0.24)		−0.14 (−0.79, 0.51)	
		Time spent on the unit	0.02 (−0.03, 0.08)		−0.03 (−0.09, 0.03)		0.04 (−0.11, 0.19)	

Regression coefficient (*β*) with 95% confidence interval (CI), and total adjusted coefficient of determination (*r^2^*) were evaluated using regression analysis. The differences in body composition results obtained from DXA and BIA were calculated as ΔFFM, ΔFM, and ΔBF%. None of the values in the table are statistically significant. FFM: Fat-free mass, FM: Fat mass, BF%: Body fat percentage, TBW%: Total body water percentage, DXA: Dual-energy X-ray absorptiometry, BIA: Bioelectrical impedance analysis.

**Table 3:** Association between time spent on the inpatient specialist unit and the changes in DXA and BIA derived body composition measures from T1 to T2.

	Time Spent on the Unit	
	Adjusted *r*^2^	*β* (95% CI)
DXA		
FFM_(T2–T1)_	0.33 **	0.09 ** (0.04, 0.15)
FM_(T2–T1)_	0.43 ***	0.12 *** (0.06, 0.17)
BF%_(T2–T1)_	0.73 ***	0.16 *** (0.12, 0.20)
BIA		
FFM_(T2–T1)_	0.34	0.10 ** (0.05, 0.16)
FM_(T2–T1)_	0.38 ***	0.11 *** (0.05, 0.17)
BF%_(T2–T1)_	0.22 **	0.16 ** (0.04, 0.28)

Regression coefficient (*β*) with 95% confidence interval, and adjusted coefficient of determination (*r*^2^) were evaluated using regression analysis. The changes in body composition results from T1 to T2 obtained by DXA and BIA were calculated. FFM: Fat-free mass, FM: Fat mass, BF%: Body fat percentage, DXA: Dual-energy X-ray absorptiometry, BIA: Bioelectrical impedance analysis. ** *p* < 0.01, *** *p* < 0.001.

## Data Availability

Data described in the manuscript, and analytical code will be made available on 31 December 2021 via the Open Science Framework (https://osf.io/v8yce/).

## Supplementary Materials

The following are available online at https://www.mdpi.com/article/10.3390/ijerph182111388/s1, Figure S1: Flow diagram of the study participants, Figure S2: Body composition measures obtained from DXA and BIA. Figure S3: Relationship between differences and means of FFM and FM obtained from DXA and BIA, Table S1: Proportion of patients with BIA estimates of body composition above the defined clinically relevant minimum values. Table S2: Correlation, concordance and Bland-Altman agreement between DXA and BIA estimates of body composition. Table S3: Association between DXA and BIA derived changes in body composition measures from T1 to T2.
