# Supplementary material for: Comparison of Dual-Energy X-ray Absorptiometry and Bioelectrical Impedance Analysis in the Assessment of Body Composition in Women with Anorexia Nervosa upon Admission and Discharge from an Inpatient Specialist Unit
Source: Int J Environ Res Public Health. 2021 Oct 29;18(21):11388. doi: 10.3390/ijerph182111388 (PMC8583076; doi:10.3390/ijerph182111388)
Supplement: Supplementary file 1 [file ijerph-18-11388-s001.zip › ijerph-1396423-supplementary.pdf]

On-line Supplementary Material

Journal: International Journal of Environmental Research and Public Health, Special Issue on "Psychology of Environmental Research and Public Health"

Title: Comparison of dual-energy X-ray absorptiometry and bioelectrical impedance analysis in the assessment of body composition in women with anorexia nervosa upon admission and discharge from an inpatient specialist unit

Authors: Afrouz Abbaspour, Kylie K. Reed, Christopher Hübel, Emily C. Bulik-Sullivan, Quyen Tang, Cynthia M. Bulik, and Ian M. Carroll

Correspondence: Afrouz.Abbaspour@ki.se; Postal address: C8 Medicinsk epidemiologi och biostatistik, C8 MEB Bulik Projektgrupp, 171 77 Stockholm; Tel: +46 70 4353664

Figure S1. Flow diagram of the study participants

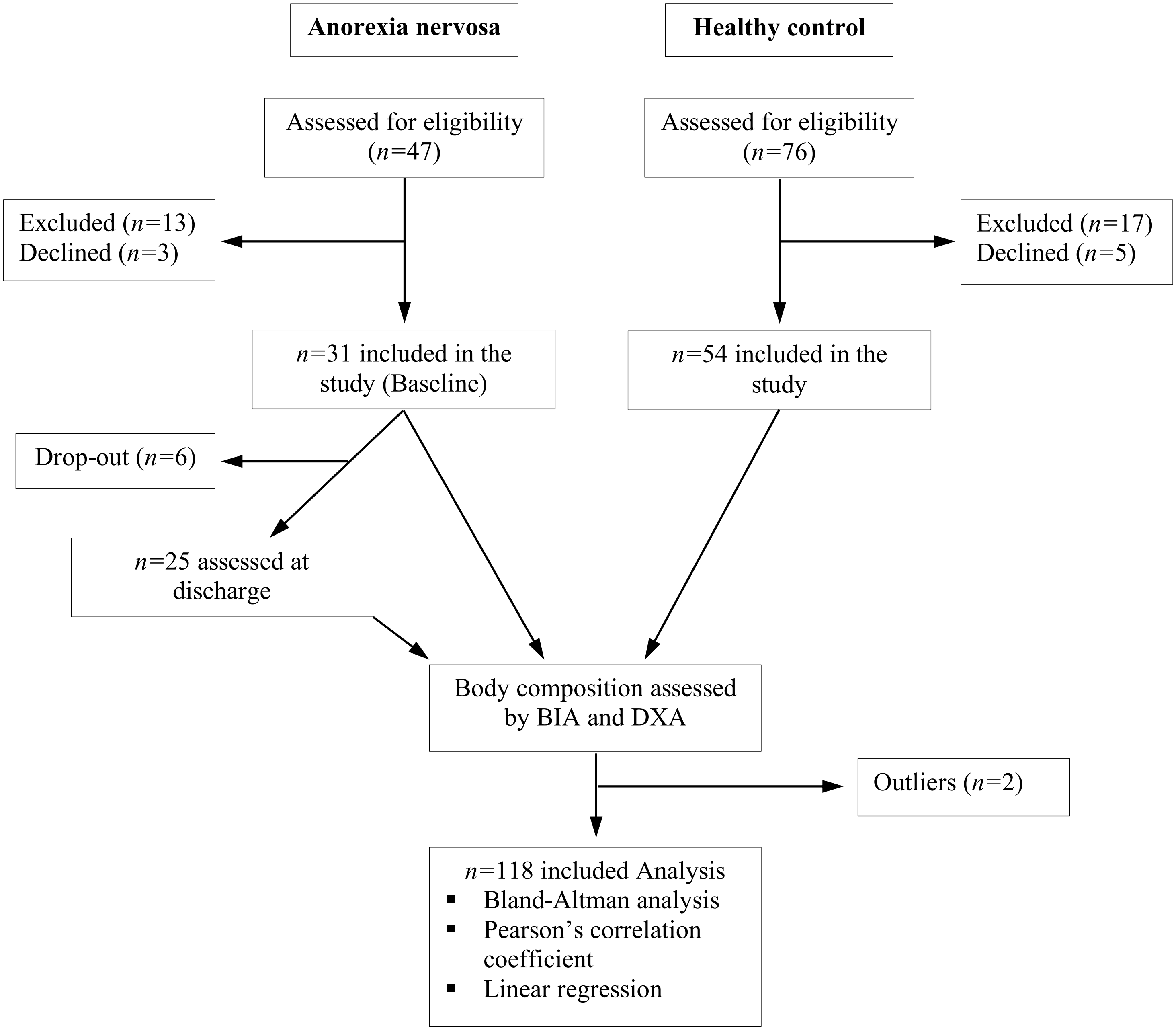

All measurements were recorded for patients with anorexia nervosa at baseline (i.e., between 1-15 days [mean 4 days] following informed consent; *n*=31) and at discharge (*n*=25). The range for duration of inpatient treatment was 3 to 75 days with a median of 21 days.

Figure S2- Body composition measures obtained from DXA and BIA

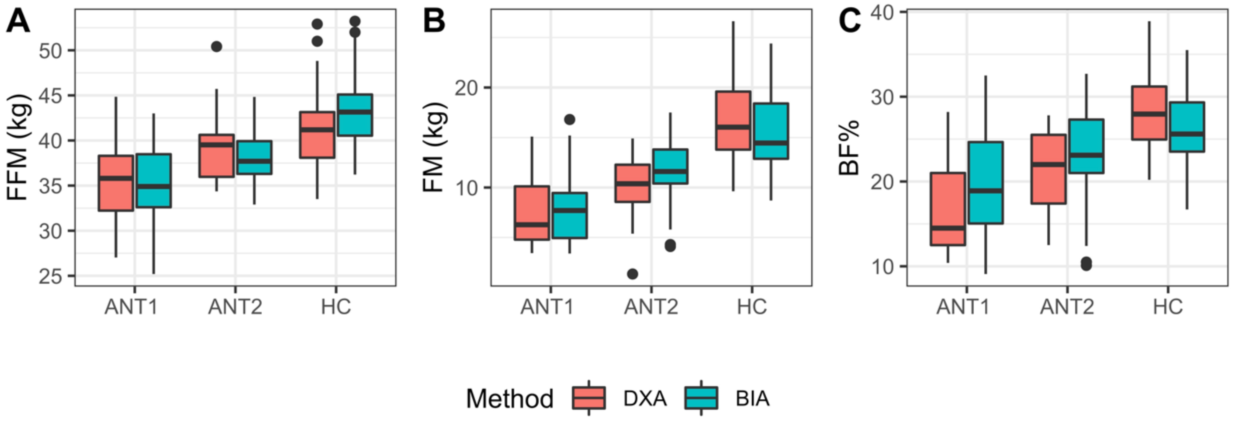

Boxplots indicating FFM **(A)**, FM **(B)**, and BF% **(C)** in ANT1, ANT2, and HC as measured by dual-energy X-ray absorptiometry (DXA) and bioelectrical impedance analysis (BIA). FFM: Fat-free mass, FM: Fat mass, BF%: Body fat percentage.

Figure S3- Relationship between differences and means of FFM and FM obtained from DXA and BIA

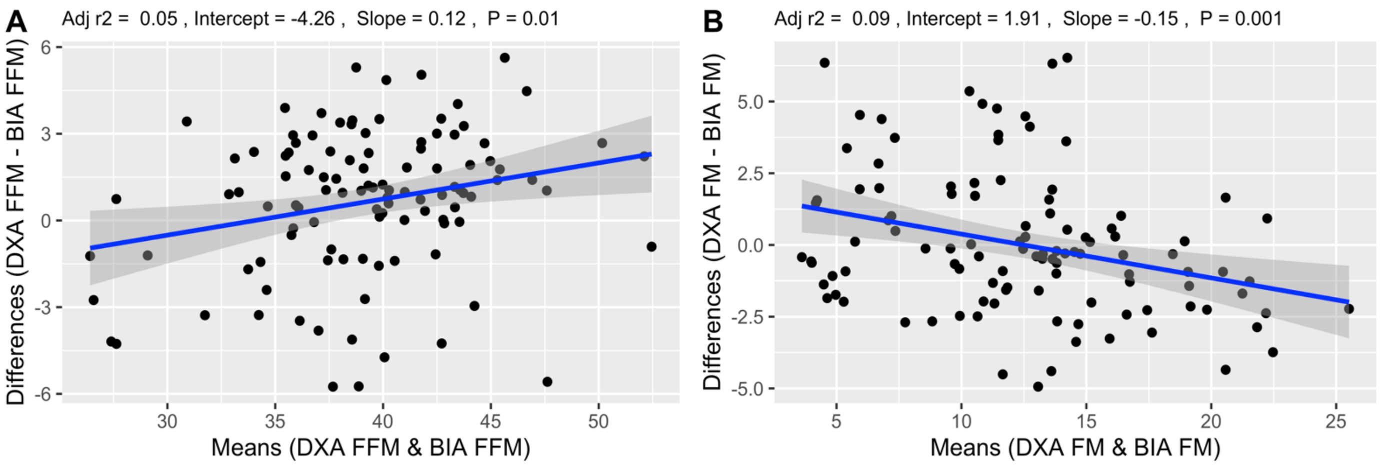

Relationship between differences and means of FFM and FM derived from DXA and BIA across the whole sample (ANT1, ANT2, and HC combined) was assessed using linear regression. Adj r^2: Adjusted r squared, FFM: Fat-free mass, FM: Fat mass.

Table S1. Proportion of patients with BIA estimates of body composition above the defined clinically relevant minimum values.

	ANT1	ANT2
**% Above Min FFM**	84	80
**% Above Min FM**	100	88
**% Above Min BF**	100	84

Clinically relevant minimum body composition values were obtained from meta-analysis by Hübel et al. For ANT1, Min FFM = 28.4, Min FM = 0.5, and Min BF%= 2.4 and for ANT2, Min FFM = 36.2, Min FM = 7.2, Min BF% = 17.3. Proportion of individuals with values above the defined clinically relevant Min values were estimated for each BIA equation at baseline (ANT1) and at discharge from an inpatient specialist unit (ANT2). FFM: Fat-free mass, FM: Fat mass, BF%: Body fat percentage.

Table S2. Correlation, concordance and Bland-Altman agreement between DXA and BIA estimates of body composition.

	ANT1	ANT2	HC
DXA vs BIA FFM (kg)			
Pearson r	0.86 ***	0.75 ***	0.92 ***
Lin ρc (95% CI)	0.85 (0.72, 0.93)	0.69 (0.46, 0.84)	0.81 (0.71, 0.87)
Mean bias (95% CI)	-0.46 (-1.40, 0.48)	-0.86 (-1.91, 0.19)	2.03 (1.58, 2.47)
ULoA (95% CI)	4.57 (2.94, 6.20)	4.11 (2.30, 5.92)	5.16 (4.39, 5.92)
LLoA (95% CI)	-5.49 (-7.12, -3.86)	-5.83 (-7.64, -4.02)	-1.10 (-1.86, -0.34)
DXA vs BIA FM (kg)			
Pearson r	0.77 ***	0.70 ***	0.91 ***
Lin ρc (95% CI)	0.73 (0.53, 0.85)	0.64 (0.36, 0.81)	0.86 (0.78, 0.91)
Mean bias (95% CI)	1.01 (0.08, 1.93)	1.49 (0.43, 2.56)	-1.32 (-1.77, -0.87)
ULoA (95% CI)	5.95 (4.35, 7.54)	6.54 (4.70, 8.38)	1.82 (1.06, 2.60)
LLoA (95% CI)	-3.93 (-5.53, -2.33)	-3.55 (-5.39, -1.71)	-4.47 (-5.23, -3.70)
DXA vs BIA BF (%)			

Pearson *r*	0.54 ***	0.50 **	0.78 ***
Lin ρc (95% CI)	0.49 (0.2, 0.7)	0.47 (0.13, 0.71)	0.69 (0.54, 0.80)
Mean bias (95% CI)	2.26 (0.05, 4.46)	1.66 (-0.59, 3.90)	-2.29 (-3.13, -1.46)
ULoA (95% CI)	14.03 (10.22, 17.83)	12.32 (8.43, 16.20)	3.57 (2.14, 5.01)
LLoA (95% CI)	-9.51 (-13.32, -5.71)	-9.00 (-12.89, -5.11)	-8.16 (-9.60, -6.73)

Correlation and concordance coefficients between BIA and DXA measures of body composition in ANT1, ANT2, and HC were estimated using Pearson's and Lin's method, respectively. Mean bias (95% CI) and limits of agreements (95% CI) between BIA relative to DXA (as the reference method) were calculated using Bland-Altman agreement analysis. BF%: FFM: Fat-free mass, FM: Fat mass, Body fat percentage, *r:* Pearson's correlation coefficient*,* ρc: Concordance correlation coefficient of Lin, LLoA: Lower limit of agreement, ULoA: Upper limit of agreement, CI: Confidence interval, $^{**}p < 0.01$, $^{***}p < 0.001$.

Table S3. Association between DXA and BIA derived changes in body composition measures from T1 to T2.

Model	Independent variables	Dependent variables	*β* (95% CI)	Total adjusted r^2
1	BIA FFM $_{(T2-T1)}$	DXA FFM $_{(T2-T1)}$	0.75 *** (0.51, 0.99)	0.63
	BIA FM $_{(T2-T1)}$	DXA FM $_{(T2-T1)}$	0.83 *** (0.59, 1.08)	0.67
	BIA BF% $_{(T2-T1)}$	DXA BF% $_{(T2-T1)}$	0.30 ** (0.09, 0.52)	0.24
2	BIA FFM $_{(T2-T1)}$	DXA FFM $_{(T2-T1)}$	1.37 *** (0.82, 1.92)	0.72
	Age		-0.09 (-0.26, 0.09)	
	TBW%		-0.03 (-0.23, 0.16)	
	Time spent on the unit		0.05 (-0.05, 0.14)	
	BIA FM $_{(T2-T1)}$	DXA FM $_{(T2-T1)}$	1.13 *** (0.64, 1.62)	0.80
	Age		0.09 (-0.11, 0.29)	
	TBW%		0.02 (-0.20, 0.25)	
	Time spent on the unit		-0.06 (-0.22, 0.1)	
	BIA BF% $_{(T2-T1)}$	DXA BF% $_{(T2-T1)}$	0.04 (-0.14, 0.23)	
	Age		-0.14 * (-0.28, -0.001)	
	TBW%		-0.05 (-0.10, 0.21)	0.84
	Time spent on the unit		0.18 ** (0.07, 0.29)	

Regression coefficient (*β*) with 95% confidence interval, and adjusted coefficient of determination (r^2) were evaluated using regression analysis. The changes in body composition results from T1 to

T2 were compared between DXA and BIA. Model 2 uses age, TBW% and time spent on the unit as covariates. FFM: Fat-free mass, FM: Fat mass, BF%: Body fat percentage, TBW%: Total body water percentage, DXA: Dual-energy X-ray absorptiometry, BIA: Bioelectrical impedance analysis. $^{*}p < 0.05$, $^{**}p < 0.01$, $^{***}p < 0.001$.

References

Hübel C, Yilmaz Z, Schaumberg KE, Breithaupt L, Hunjan A, Horne E, García-González J, O'Reilly PF, Bulik CM, Breen G. Body composition in anorexia nervosa: Meta-analysis and meta-regression of cross-sectional and longitudinal studies. Int J Eat Disord. 2019;52:1205–23
